# Validity of Web-Based Self-Reported Weight and Height: Results of the Nutrinet-Santé Study

**DOI:** 10.2196/jmir.2575

**Published:** 2013-08-08

**Authors:** Camille Lassale, Sandrine Péneau, Mathilde Touvier, Chantal Julia, Pilar Galan, Serge Hercberg, Emmanuelle Kesse-Guyot

**Affiliations:** ^1^Université Paris 13, Sorbonne Paris Cité, UREN (Nutritional Epidemiology Research Unit), Inserm (U557), Inra (U1125), CnamBobignyFrance; ^2^Public Health DepartmentHôpital AvicenneBobignyFrance

**Keywords:** anthropometry, body weight, obesity, self-report, weights and measures, validation studies

## Abstract

**Background:**

With the growing scientific appeal of e-epidemiology, concerns arise regarding validity and reliability of Web-based self-reported data.

**Objective:**

The objectives of the present study were to assess the validity of Web-based self-reported weight, height, and resulting body mass index (BMI) compared with standardized clinical measurements and to evaluate the concordance between Web-based self-reported anthropometrics and face-to-face declarations.

**Methods:**

A total of 2513 participants of the NutriNet-Santé study in France completed a Web-based anthropometric questionnaire 3 days before a clinical examination (validation sample) of whom 815 participants also responded to a face-to-face anthropometric interview (concordance sample). Several indicators were computed to compare data: paired t test of the difference, intraclass correlation coefficient (ICC), and Bland–Altman limits of agreement for weight, height, and BMI as continuous variables; and kappa statistics and percent agreement for validity, sensitivity, and specificity of BMI categories (normal, overweight, obese).

**Results:**

Compared with clinical data, validity was high with ICC ranging from 0.94 for height to 0.99 for weight. BMI classification was correct in 93% of cases; kappa was 0.89. Of 2513 participants, 23.5% were classified overweight (BMI≥25) with Web-based self-report vs 25.7% with measured data, leading to a sensitivity of 88% and a specificity of 99%. For obesity, 9.1% vs 10.7% were classified obese (BMI≥30), respectively, leading to sensitivity and specificity of 83% and 100%. However, the Web-based self-report exhibited slight underreporting of weight and overreporting of height leading to significant underreporting of BMI (*P*<.05) for both men and women: –0.32 kg/m^2^ (SD 0.66) and –0.34 kg/m^2^ (SD 1.67), respectively. Mean BMI underreporting was –0.16, –0.36, and –0.63 kg/m^2^ in the normal, overweight, and obese categories, respectively. Almost perfect agreement (ie, concordance) was observed between Web-based and face-to-face report (ICC ranged from 0.96 to 1.00, classification agreement was 98.5%, and kappa 0.97).

**Conclusions:**

Web-based self-reported weight and height data from the NutriNet-Santé study can be considered as valid enough to be used when studying associations of nutritional factors with anthropometrics and health outcomes. Although self-reported anthropometrics are inherently prone to biases, the magnitude of such biases can be considered comparable to face-to-face interview. Web-based self-reported data appear to be an accurate and useful tool to assess anthropometric data.

## Introduction

Overweight and obesity have reached pandemic proportions and it is considered as one of the major public health issues by the World Health Organization (WHO) [1-3]. Excess body weight is a major risk factor of various chronic conditions, such as hypertension, type 2 diabetes, cardiovascular diseases, and some cancers [4].

Body mass index (BMI), defined as weight (kg) divided by squared height (m^2^), is highly correlated to excess fat mass. It is commonly used to classify overweight and obesity in adults: overweight excluding obesity (BMI 25-29 kg/m^2^) and obesity (BMI ≥30 kg/m^2^) [1]. In large-scale multicentric epidemiologic studies, self-reporting of weight and height is usually used because of substantial logistic and cost savings as compared with direct measures by trained technicians. In that context, self-reporting is actually the more effective and manageable way to collect anthropometric data in large samples up to tens of thousands of participants.

However, it is acknowledged that self-reported height and weight are biased proxies of the true measures. Indeed, bias between self-reported and measured anthropometrics has been widely described in the scientific literature, in many American and European studies [5-13]. Generally, weight is underreported whereas height is overreported, [5,12] leading to an underestimation of BMI and a misclassification in BMI categories, although errors vary according to sex, age, education, and socioeconomic characteristics [8,10,11,14,15]. Moreover, biases are likely differential with a relationship between magnitude of bias and measured BMI: underweight participants tend to overreport whereas overweight participants tend to underreport their weight [16]. This phenomenon is partly explained by social desirability, which can be further influenced by the method of data collection [5,7,17,18]. For example, evidence for social desirability bias was observed in the Canadian Community Health Survey, which studied the difference between face-to-face and telephone self-reported anthropometrics and showed that obesity prevalence in the face-to-face group was significantly higher than in in the phone group (18% and 13%, respectively) [18]. This suggests a tendency to underreport weight to attempt to construct favorable images in the eyes of others, to get closer to a socially ideal weight when the interviewer cannot visually assess it [19]. In that context, it is of interest to assess whether Web-based self-report would lead to the same discrepancy with face-to-face compared to what is observed between telephone and face-to-face self-report.

A novel approach for large-scale epidemiologic studies lies in the use of Internet to administer Web-based questionnaires [20-25], which is recognized as the new promising field of e-epidemiology. A key advantage of a Web-based epidemiologic study is the substantial logistic and cost savings compared with traditional data collection (pencil and paper questionnaires, face-to-face interviews). Other features, such as data management improvement and simplification, flexibility, and recruitment of large samples, can be achieved with e-epidemiology.

In the NutriNet-Santé study, comparison of self-reported weight and height in a Web-based anthropometric questionnaire with the traditional paper form of the same questionnaire showed satisfying results, which were published elsewhere [26].

To date, only 1 study focused on assessing validity of Web-based self-reported weight compared with direct measure [27]. However, this study did not provide insight on the validity of Web-based self-reported height or BMI because height was not measured. To the best of our knowledge, the comparison between Web-based and face-to-face self-reported anthropometrics has never been published.

The objectives of the present study were to (1) assess the validity of Web-based self-reported weight, height, and resulting BMI compared with measured data in a subsample of the NutriNet-Santé study, and (2) evaluate the concordance (ie, agreement) between Web-based self-reported anthropometrics and face-to-face declaration. We hypothesized that (1) we would observe underreporting of BMI with the Web-based questionnaire compared with the gold standard (ie, clinical measurement), and (2) social desirability in front of the computer would be less important than on the phone compared with the face-to-face interview.

## Methods

### The NutriNet-Santé Study

The present analyses were carried out on a subsample of the NutriNet-Santé study, an ongoing Web-based prospective cohort study launched in France in May 2009 [28] aiming to investigate the associations between nutrition and health and to study the role of various determinants (sociodemographic, economic, biochemical, cognitive, etc) of dietary behavior and nutritional status. Recruitment of adult volunteers (aged ≥18 years) through multimedia campaigns is to be carried out for 5 years with a planned additional follow-up of 10 years.

Briefly, at inception, participants complete a set of Web-based questionnaires assessing socioeconomic and sociodemographic conditions, dietary intake, physical activity, anthropometrics, lifestyle, and health status [28]. Each month, participants are invited to fill in complementary optional questionnaires related to determinants of dietary behavior and nutritional and health status. The anthropometric questionnaire is repeated every 6 months.

Moreover, participants are invited to attend one of the specific health centers involved in the study, located in various French cities. During the visit, they undergo blood and urine sampling and a clinical examination including anthropometric measurements. Height is measured by a trained technician with a wall-mounted stadiometer without shoes to the nearest 0.5 cm [29]. Weight is measured with a calibrated scale (body composition analyzer BC-418MA, TANITA, Tokyo, Japan) to the nearest 0.1 kg, with participants wearing indoor clothes, without shoes, socks, or stockings. Height is entered manually into the TANITA software, and then weight is measured, with the data sent automatically to the database through a secured interface. Results are checked with the participant allowing for detection of any typing errors regarding height. Complete information about the NutriNet-Santé study design can be found elsewhere [28].

This study was approved by the International Research Board of the French Institute for Health and Medical Research (IRB Inserm no: 0000388FWA00005831) and the French National Information and Citizen Freedom Committee (CNIL no: 908450 and no: 909216). The collection of biological samples and clinical data was approved by the Consultation Committee for the Protection of Participants in Biomedical Research (C09-42 on May 5, 2010) and the French National Information and Citizen Freedom Committee (CNIL no: 1460707).

### Validation and Concordance Samples

To validate the self-reported anthropometrics, a random subsample of the participants with a scheduled clinical examination were invited to fill in a Web-based anthropometric questionnaire 3 days before their appointment at the health center. This minimizes weight variations because of a long time lag between reported and measured weight. The validation study started in November 2011 and ended in July 2012. All participants with a scheduled visit in this time range were invited to fill in the anthropometric questionnaire. A total of 2513 participants completed the questionnaire 3 days before and had attended the subsequent clinical visit. This constitutes the validation sample.

Among them, some randomly assigned participants were asked by the trained technicians to declare their height and weight on the day of the examination, before being measured. The concordance study started in February 2012. By July 2012, a total of 815 participants had provided Web-based weight and height 3 days before and in a face-to-face interview, constituting the concordance sample. We chose to stop inclusions and start the analyses in July 2012 because it provided a good balance between an acceptable sample size as reviewed [5] and a reasonable study duration.

### Covariates

Socioeconomic variables were collected at study baseline. Education referred to the highest achieved level (primary school, secondary school, high school diploma, university bachelor degree or less, university graduates with higher than bachelor degree) and was further regrouped into 3 categories (up to high school diploma, university bachelor degree or less, university graduates with higher than bachelor degree); occupational category was defined according to the current job or the last job held for unemployed or retired individuals (never employed, self-employed, farmers, manual workers, intermediate professions, managerial/professional staff). Monthly household income and household composition (marital status, number and age of children) were also reported, which allowed calculating monthly income per household unit (in euros) by using a standardized algorithm [30] and were categorized in quartiles. Tobacco use (current, former, never smoked), and marital status were also used as covariates.

Leisure time physical activity (LTPA) was assessed by the International Physical Activity Questionnaire (IPAQ) [31,32] and classes of physical activity were defined as recommended [33] in low, medium, and high LTPA categories. LTPA data are collected each year in the NutriNet-Santé study, so the most recent report was used.

### Statistical Analysis

For comparison to self-declared data, measured weight was rounded to the nearest kilogram and height to the nearest centimeter. Log-transformation was applied to height, weight, and resulting BMI to improve normality. BMI was categorized as normal (BMI<25 kg/m^2^), overweight excluding obesity (BMI 25-29 kg/m^2^), and obese (BMI ≥30 kg/m^2^). Throughout this paper, *overweight* refers to overweight excluding obesity, unless otherwise stated.

Population characteristics (sex, age, socioeconomic status, tobacco use, LTPA, and anthropometrics) were compared between the validation and concordance samples and with the entire NutriNet-Santé cohort by *t* tests and chi-square (χ^2^) tests.

A summary of the indicators used for validation and concordance analyses is provided in Multimedia Appendix 1.

### Validation Analysis

Several statistical procedures were used to assess the validity of Web-based self-reported anthropometrics by comparing them to the reference values measured by the technician. The difference between self-reported and measured weight, height, and resulting BMI were calculated. *P* value referred to paired *t* test (on log-transformed variables). To assess agreement between self-reported and measured values, a random effect model was performed to estimate intraclass correlation coefficient (ICC) (2,1) as proposed by Shrout and Fleiss [34] using the SAS macro %INTRACC [35]. We also used the Bland-Altman method [36]: for each variable (log transformed), the difference self-reported minus measured was plotted against the average (self-reported + measured)/2, providing mean agreement and 95% limits of agreement (LOA) defined as mean agreement ±2 SD of the difference. Because results were antilogged after analysis, the mean agreement and LOA are given as ratio of self-reported to measured values [37,38]. A mean agreement of 100% represents exact agreement, otherwise there is systematic bias. If agreement is >100%, it indicates that, on average, participants overreported, whereas <100% indicates underreporting compared to the measure. The slope of average of methods regressed on the difference between methods was also estimated to test the existence of proportional bias although the Bland-Altman method does not adequately distinguish between fixed and proportional bias [39]. To further investigate the influence of socioeconomic and lifestyle factors (BMI category, age, sex, LTPA, smoking status, education level, level of income, occupation), bivariate and multivariate regression analyses were used, considering the difference between self-reported and measured height, weight, or BMI as the dependent variables.

Percentage of agreement between self-reported and measured categories of BMI were calculated and the degree of misclassification was assessed through weighted kappa coefficient. McNemar tests were carried out for the binary variables (1) overweight including obesity and (2) obese. Sensitivity and specificity for overweight and obese were also calculated as true positives/(true positives + false negatives) and true negatives/(true negatives + false positives), with the true measure being the clinical data.

### Concordance Analyses

The same procedures were used for the concordance study between self-reported Web-based questionnaire and face-to-face interview, namely paired *t* test of the difference between Web and face-to-face values, ICC, Bland–Altman regression and LOA, percentage of agreement, and weighted kappa coefficient.

### Sensitivity Analyses

Because participants who answered the Web-based anthropometric questionnaire 3 days before attending the visit knew that they would be measured, this could lead to overagreement between self-reported and measured data. To overcome this potential bias, we performed the following sensitivity analyses: a second validity sample included participants who filled in the regular Web-based anthropometric questionnaire (available every 6 months) within 2 months before attending the visit. The visit was not necessarily scheduled at time of completion; hence, participants were unaware of an upcoming measurement. A time lag of a maximum 2 months was chosen to limit actual weight variations. The second validity sample consisted of 2078 participants. Among them, a second concordance sample of 233 participants was drawn that had available data from the face-to-face declaration.

All statistical tests were 2-sided and *P*<.05 was considered significant. All statistical analyses were performed using SAS software ver 9.1 (SAS Institute Inc, Cary, NC, USA).

## Results

### Population Characteristics

The characteristics of the entire NutriNet-Santé cohort and of the validity and concordance samples are presented in Table 1. There were no significant differences between the validity and concordance samples regarding age, education, occupation, smoking status, and LTPA. Participants in the validity sample were less often women, significantly older, more physically active, less likely to be smokers, and more likely to live with a partner and to have a higher level of income than the entire cohort (all *P* values <.001). Web-based self-reported anthropometrics showed no significant difference between the validity sample and the cohort, except for a slightly higher height (*P*=.003).

### Validity

Men and women underreported their weight by –0.40 kg (SD 1.45) and –0.52 kg (SD 1.42), respectively, and overreported their height by 0.61 cm (SD 1.40) and 0.55 cm (SD 2.66), leading to an underreporting of BMI of –0.32 kg/m^2^ (SD 0.66) for men and –0.34 kg/m^2^ (SD 1.67) for women (all *P*<.001) (Multimedia Appendix 2). No difference was observed between men and women for BMI, height (*t* test *P* values >.05), and weight (*P*=.05).

**Figure 1:**
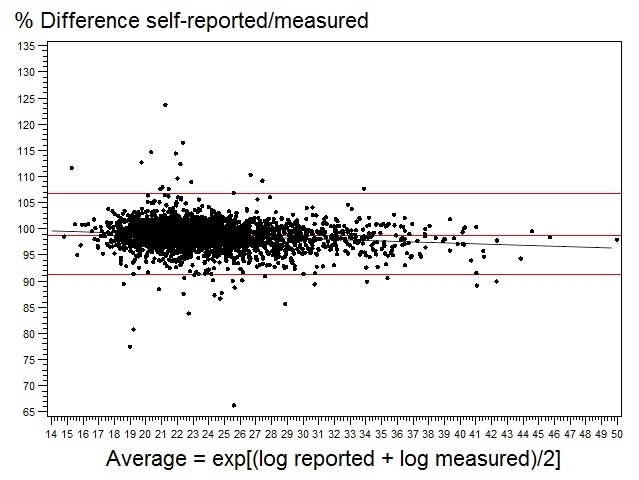
Bland - Altman plot of self-reported versus measured values of BMI, NutriNet-Santé study, 2012, France. Horizontal lines represent the % mean difference and 95% limits of agreement.

Validity of continuous variables is presented in Table 2. Overall, agreement was high between self-reported and measured anthropometric data with ICC ranging from 0.94 (height) to 0.99 (weight). However, a systematic bias was observed for each variable because percent mean agreement was significantly different from 100%, indicating underreporting of weight and BMI and overreporting of height. The LOA were wider for BMI than for height and weight. For approximately 95% of cases, self-reported BMI differed from measured BMI by 8.9% less than to 6.7% greater than the real value (LOA are provided compared to the reference, ie, 100%, but are symmetrical in relation to the mean of agreement value, here 98.6%; Figure 1).

To investigate determinants of differential bias, we regressed the difference between self-reported and measured BMI values on covariates. BMI category showed a significant effect (crude and adjusted for covariates: sex, age, LTPA, occupation, education, and smoking). BMI underreporting was –0.16, –0.36, and –0.63 kg/m^2^ among normal, overweight, and obese participants, respectively, in the adjusted model. Weight underreporting was significantly associated with BMI category (more underreporting among obese and overweight vs normal) and sex (women underreported more than men). Height overreporting was positively associated with BMI category (more overreporting among obese and overweight vs normal) and age. Crude differences by sex, across BMI and age categories are reported in Multimedia Appendix 2.

Table 3 shows an agreement of 93.2% between BMI categories and a weighted kappa of 0.89. The overweight proportion was 2.2 percentage points less when estimated from self-reported than from clinical data (23.5% vs 25.7%) and 1.7 points less for obesity (9.1% vs 10.7%). The difference was statistically significant according to the McNemar test (*P*<.001). Regarding detection of obesity, out of 270 truly obese participants, 45 were not classified obese with the self-report (false negative) whereas 225 were well-detected (true positive), leading to a sensitivity of 83.3% and a specificity of 99.9%. Regarding detection of overweight including obesity (BMI≥25), 97 participants were false negative and 818 true positive, leading to a sensitivity of 87.9% and a specificity of 99.1%.

### Concordance

As shown in Table 4, mean agreement between Web-based and face-to-face values was almost perfect; the difference was not significant and ICCs were 1.00, 0.96, and 0.98 for weight, height, and BMI, respectively.

As presented in Table 5, agreement in BMI categories was also very strong with 98.5% of the participants similarly classified in BMI classes. The weighted kappa was 0.97 and difference in overweight classification was not significant, but it was significant for obesity (*P=*.01).

**Table 1:** Characteristics of the validation study sample (N=2513) and the concordance study sample (n=815) from the NutriNet-Santé Study, 2012, France.

Participants’ characteristics		Validity sample (V) n=2513	Concordance sample (C)^a^	NutriNet-Santé cohort (CO) n=115,784	*P* value^b^	
					V vs CO	C vs CO
Age (years), mean (SD)		53.8 (13.3)	53.6 (13.0)	45.1 (14.5)	<.001	<.001
**Weight (kg)** ^c^						
	Mean (SD)	66.8 (13.2)	66.5 (13.4)	67.3 (15.1)	.06	.11
	Median (IQR)	65 (57-75)	64 (57-74)	64 (57-75)		
**Height (cm)** ^c ^						
	Mean (SD)	166.3 (8.3)	165.7 (8.5)	166.8 (8.5)	.003	.001
	Median (IQR)	165 (160-172)	165 (160-170)	166 (161-172)		
**BMI (kg/m^2^)** ^c^						
	Mean (SD)	24.1 (4.3)	24.2 (4.4)	24.2 (5.2)	.49	.95
	Median (IQR)	23.3 (21.1-26)	23.5 (21.2-26.2)	23.1 (20.8-26.2)		
Female, n (%)		1835 (73.0)	606 (74.4)	90,382 (78.1)	<.001	.01
Living with a partner, n (%)		1860 (74.0)	607 (74.5)	82,480 (71.2)	.001	.04
**BMI (kg/m^2^),** ^d^ **n (%)**					.26	.61
	Normal (<25 kg/m^2^)	1604 (63.8)	513 (62.9)	76,879 (67.2)		
	Overweight (25-29 kg/m^2^)	643 (25.6)	210 (25.8)	25,396 (22.2)		
	Obese (≥30 kg/m^2^)	266 (10.6)	92 (11.3)	12,125 (10.6)		
**Education, n (%)**					.44	.96
	Primary school	78 (3.2)	22 (2.8)	3854 (3.4)		
	Secondary school	491 (20.1)	156 (19.8)	19,971 (17.6)		
	High school diploma	374 (15.3)	113 (14.3)	20,557 (18.1)		
	University < bachelor degree	746 (30.5)	264 (33.4)	33,362 (29.5)		
	University ≥ bachelor degree	757 (31.0)	235 (29.8)	35,552 (31.4)		
**Occupational category, n (%)**					.39	.94
	Never employed	55 (2.2)	18 (2.2)	6646 (5.7)		
	Self-employed. farmers	101 (4.0)	33 (4.1)	3951 (3.4)		
	Manual workers	53 (2.1)	21 (2.6)	3509 (3.0)		
	Intermediate professions	1372 (54.6)	436 (53.5)	65,223 (56.3)		
	Managerial/professional	932 (37.1)	307 (37.7)	36,455 (31.5)		
**Tobacco smoking, n (%)**					<.001	.001
	Current smoker	241 (9.6)	86 (10.5)	2079 (18.0)		
	Former smoker	999 (39.7)	320 (39.3)	38,324 (33.1)		
	Never smoker	1273 (50.7)	409 (50.2)	5667 (48.9)		
**Physical activity level,** ^d^ **n (%)**					<.001	.79
	Low	498 (20.3)	176 (22.1)	27,212 (25.6)		
	Medium	1002 (40.8)	300 (37.6)	44,239 (41.7)		
	High	954 (38.9)	322 (40.3)	34,695 (32.7)		

**Level of income (€/unit of consumption), n (%)**					<.001	<.001
	Don’t want to answer	261 (10.4)	91 (11.2)	14,929 (13.5)		
	<1257	302 (12.0)	112 (13.7)	23,511 (21.3)		
	1257-1835	508 (20.2)	166 (20.4)	23,606 (21.4)		
	1835-2700	674 (26.8)	225 (27.6)	24.,329 (22.1)		
	>2700	768 (30.6)	221 (27.1)	23.,849 (21.6)		

^a^No significant difference was observed between the validity and concordance samples (all *P* values >.05 for chi-square tests or *t* test for age).

^b^ *P* value for *t* test or Mantel–Haenszel chi-square test as appropriate.

^c^ *t* tests on the log-transformed variables.

^d^Reduced sample size because of missing values; validity sample: n=2454 for physical activity level; concordance sample: n=798 for physical activity level; cohort: n=114,400 for BMI, n=113,296 for education, n=106,146 for physical activity level.

**Table 2:** Validity indicators of weight, height, and body mass index (BMI) including intraclass correlation coefficient (ICC) between the Web-based self-report and measurement at the clinical examination, Bland–Altman mean agreement, and limits of agreement (LOA) from the NutriNet-Santé Study, 2012, France (N=2513).

Anthropometric variables	Web-based		Measured		Difference		*P* ^a^	ICC^b^		% mean agreement^c^		% LOA^d^	
	Mean	SD	Mean	SD	Mean	SD		ICC	95% CI	%	95% CI	Lower limit	Upper limit
Weight (kg)	66.84	13.60	67.33	13.74	–0.49	1.43	<.001	0.99	0.99, 0.99	99.28	99.20, 99.37	95.11	103.64
Height (cm)	166.30	8.48	165.73	8.32	0.56	2.39	<.001	0.94	0.94, 0.95	100.33	100.27, 100.40	97.06	103.72
BMI (kg/m^2^)	24.12	4.44	24.46	4.41	–0.34	1.47	<.001	0.97	0.97, 0.97	98.61	98.47, 98.77	91.12	106.74

^a^ *P* value of the paired *t* test of difference of log-transformed variable.

^b^ICC(2,1) calculated on log-transformed variables.

^c^Bland–Altman mean agreement (average of difference self-reported – measured). A mean agreement of 100% represents exact agreement between the 2 methods.

^d^LOA: limits of agreement of self-reported value expressed as a percent of the measured value. Because results were antilogged after analysis, the LOA are given as ratio Web:measured.

**Table 3:** Validity indicators for categorical variables including percent of similar classification and weighted kappa coefficient for overweight and obesity classification between the Web-based declaration and reference measurement at clinical examination from the NutriNet-Santé Study, 2012, France (N=2513).

Categorical anthropometric variable	Web-based n=2513		Measured n=2513		Agreement (%)		Weighted kappa^a^		*P* ^b^	Sensitivity^c,d^		Specificity^c,e^	
	n	%	n	%	%	95% CI	κ	95% CI		%	95% CI	%	95% CI
BMI classification					93.2	92.2, 94.1	0.89	0.88, 0.91					
Normal (BMI<25)	1695	67.45	1 598	63.59									
Overweight (BMI 25-29.9)	590	23.48	645	25.67					<.001	87.9	0.86, 0.90	99.1	98.7, 99.6
Obese (BMI≥30)	228	9.07	270	10.74					<.001	83.3	78.9, 87.8	99.9	99.7, 100

^a^Cicchetti–Allison weight. For a given cell in row i, column j, w_ij_=1–(|i–j|/2).

^b^ *P* value of McNemar chi-square test for binary variables: overweight including obesity (BMI≥25) yes/no and obese (BMI≥30) yes/no. A *P* value <.05 indicates significant difference between Web-based self-reporting and measurement.

^c^Sensitivity and specificity for binary variables: overweight including obesity (BMI≥25) and obese (BMI≥30).

^d^Sensitivity=true positives/(true positives + false negatives).

^e^Specificity=true negatives/(true negatives + false positives). True = clinical data.

**Table 4:** Concordance indicators for continuous variables including intraclass correlation coefficient (ICC) between Web-based and face-to-face reported data, Bland–Altman mean agreement, and limits of agreement (LOA) from the NutriNet-Santé Study, 2012, France (n=815).

Anthropometric variable	Web-based		Face-to-face		Difference		*P* ^a^	ICC^b^		% mean agreement^c^		% LOA^d^	
	Mean	SD	Mean	SD	Mean	SD		ICC	95% CI	%	95% CI	Lower limit	Upper limit
Weight (kg)	66.60	13.45	66.60	13.49	0.00	1.14	.31	0.996	0.995, 0.996	100.01	99.89, 100.14	96.46	103.69
Height (cm)	165.75	8.50	165.71	8.24	0.04	2.21	.77	0.958	0.951, 0.963	100.02	99.91, 100.12	97.13	102.98
BMI (kg/m^2^)	24.20	4.40	24.19	4.28	0.01	1.20	.78	0.979	0.976, 0.982	100.00	99.80, 100.27	93.43	107.10

^a^ *P* value of the paired *t* test of difference of log-transformed variable (Web minus face-to-face).

^b^ICC: intraclass correlation (2,1) calculated on log-transformed variables.

^c^Bland and Altman mean agreement (average of differences “Web-based minus face-to-face”). A mean agreement of 100% represents exact agreement between the 2 questionnaires.

^d^LOA: limits of agreement of Web-based self-reported value expressed as a percent of the face-to-face reported value. Because results were antilogged after analysis, the LOA are given as ratio Web-based/face-to-face.

**Table 5:** Concordance indicators for categorical variables: percent of similar classification and weighted Kappa coefficient for overweight and obesity classification between Web-based and face-to-face reported data from the NutriNet-Santé Study, 2012, France (n=815).

Categorical anthropometric variable	Web-based		Face-to-face		Agreement (%)		Weighted kappa^a^		*P* ^b^
	n	%	n	%	%	95% CI	κ	95% CI	
BMI classification					98.5	97.7, 99.4	0.97	0.96, 0.99	
Normal (BMI<25)	547	67.1	546	67.0					
Overweight (BMI 25-29.9)	193	23.7	188	23.1					1.00
Obese (BMI≥30)	75	9.2	81	9.9					.01

^a^Cicchetti–Allison weight. For a given cell in row i, column j, w_ij_=1–(|i–j|/2)

^b^ *P* value of McNemar chi-square test for binary variables: overweight including obesity (BMI>=25) yes/no and obese (BMI>=30) yes/no.

### Sensitivity Analyses

Sensitivity analyses in the second validity sample (n=2078) showed similar results as the validity sample, the validity indicators (ICC, kappa, percent agreement) were even slightly higher (Multimedia Appendix 3). However, a significant difference in weight reporting was observed in the second concordance sample (n=233): participants reported higher weight (mean 0.37, SD 1.86) and, hence, BMI (mean 0.32, SD 0.83) in the Web-based questionnaire than in the face-to-face interview. Weighted kappa was lower than the concordance sample, with a value of 0.91 (95% CI 0.86-0.95) and percent correct classification was 94%. Nevertheless, ICCs were similar, ranging from 0.98 to 0.99.

## Discussion

### Principal Finding

In the present study, we observed that Web-based self-report of anthropometrics in the NutriNet-Santé study is equivalent to a face-to-face interview. Although, as hypothesized, it is subject to bias as compared with direct measures, the bias is reasonably small and the validity indicators show good reliability of this data.

### Validity

Overall, our results showed high validity of self-reported anthropometric data compared with measured values. However, we observed a small although significant underreporting of weight and BMI and an overreporting of height, which was expected and is consistent with previous research [5]. Compared with the bias reported in the literature, the extent of misreporting in the present study (-0.49 kg for weight and 0.56 cm for height) is smaller than in most of the studies on general adult populations which show underreporting ranging from -0.1 to -6.5 kg for weight and overreporting from 0.6 to 7.5 cm for height [5]. Results of the other study assessing validity of Web-based self-reported anthropometrics showed greater underreporting of weight (-1.2 kg) and found no significant difference between men and women [27]. BMI classification is more of a concern when studying the association of nutritional factors with obesity or overweight risk. But, a correct classification of 93% and a kappa of 0.89 (which can be considered almost perfect [40]) reflect reliable and suitable results. For example, in the Adventist Health Study, correct classification in BMI categories was 83.4% (95% CI 80.9%, 85.8%) [41]. In our study, sensitivity of self-reported BMI to detect obesity was 83% and specificity was 100%, which are higher than the Adventist study (sensitivity 81%, specificity 97%), and much higher than observed in a Swiss and French community-based sample (sensitivity: 66% for men, 73% for women; specificity: 99% for both) [16]. Regarding comparability of our study population with other studies, in the Adventist study, the prevalence of self-reported obesity (27.3%) was higher than in our study (9.1%); however, the study by Dauphinot et al [16] reported exactly the same proportion of obese participants as in our study.

No difference in misreporting was observed between men and women for height, whereas it has been previously suggested that men tended to overreport their height more strongly than women [8,10,14-16,42,43], although a few studies found no difference according to sex [9,27,41]. However, we found that being a woman was a predictor of greater underreporting of weight, consistent with previous research [8,10,14-16,38,39]. Age was a significant predictor for overreporting of height, in accordance with most the studies [9,11,41,44,45]. This can be, at least partly, explained by the fact that aging is associated with a decrease in height that people might be unaware of if they are not often measured [13,19] .

Although underreporting of BMI and weight and overreporting of height was observed in every BMI category, their magnitude differed and we found that objective overweight and obesity were the strongest predictors for underreporting of weight and BMI and overreporting of height, similar to many studies [10,11,16,19,41,44,45]. Our results are very similar to those of the Adventist study [41] that showed a BMI underreporting of -0.4 kg/m^2^ in nonobese vs -0.9 kg/m^2^ in obese participants. We found lower differences between BMI categories than in the Oxford EPIC study [10] in which underreporting among normal, overweight, and obese participants was -0.6, -1.02, and -1.66 kg/m^2^ for men and -0.44, -0.96, and -1.35 kg/m^2^ for women, and in the study by McAdams et al [45] in which BMI misreporting was 0.03 (nonsignificant), -0.57, and -1.77 kg/m^2^ in normal, overweight, and obese participants, respectively. Regarding weight underreporting, our results show less difference between BMI categories than Bonn et al [27] who found underreporting of -0.9 kg in participants with BMI<25 vs -2.1 kg in overweight/obese participants. A hypothesis to explain this phenomenon lies in the social desirability concept: people are influenced by their desire to conform to perceived societal norms, and this is more important in obese participants [19].

### Concordance

Method of data collection can influence responses to surveys [46]. Several studies reported stronger underestimation of weight and BMI with telephone reporting than with face-to-face interviews [46-48]. Some hypotheses have been proposed to interpret such findings [18], including the idea that social desirability may influence reporting that cannot be visually verified [48].

Contrarily, and as hypothesized, in our study we showed almost perfect agreement between the Web-based reporting and the face-to-face interview, arguing that behind the computer screen, participants do not seem more prone to social desirability bias. This can be explained by the greater feeling of anonymity on the Web than on the telephone [48], in which the involvement is greater when the interviewer is a person rather than a computer screen. Indeed, even if the participants knew they would be weighed and measured after the face-to-face interview, this did not appear to influence what they declared.

We were aware that the Web-based reporting might be partly biased because participants theoretically knew they would be weighed a few days later; thus, limiting prevarication bias. However, the sensitivity analysis provided similar results, with even higher values of Web-based weight vs face-to-face, closer to the true measure. This shows an advantage of Web-based self-report compared with telephone interview as we previously demonstrated concerning dietary data [49].

### Strength and Limitations

The first limitation pertains to a potential underestimation of the difference between Web-based reports and measures because participants in our study knew they would attend the visit 3 days after filling in the Web-based questionnaire. However, the sensitivity analyses with data collected within 2 months before the visit showed similar results—even slightly higher validity—indicating that the difference seems not to be reduced by awareness of the upcoming examination.

Second, caution is also advised regarding the generalizability of our results. Indeed, the participants of the NutriNet-Santé study were recruited on a voluntary basis, implying that they might be particularly likely to engage in healthy behaviors; thus, a self-selection bias could have occurred in our population as in most prospective cohort studies. In particular, participants were invited to answer an anthropometric questionnaire twice a year, so they were likely to be more aware of their true weight. Further, the present validation study is subject to an additional selection bias related to the participation to the visit because some characteristics, such as age, smoking status, or LTPA, were significantly different between the validation sample and the entire cohort. However, even if some socioeconomic characteristics were different, educational level, occupation, and the main outcomes, anthropometric values, were not significantly different of the entire cohort. Also, among the participants who attended the clinical examination, those participating in the face-to-face interview were randomly allocated.

A major strength of this validation study is its originality. This is the second study assessing validity of anthropometric data collected through a Web-based tool, but we used a wider range of statistical tools that allowed analyzing the validity in more depth on a wider sample than in the recently published study [27]. This type of study is of major interest with the arising development of e-epidemiology. Also, the sample size is large and ranks among the larger validation samples published [5]. Another great strength is that the elapsed time between Web-based self-report and direct measure was controlled for, equal for every participant, and sufficiently short to avoid any true potential change in weight. Moreover, the gold standard used here, measured weight and height, was obtained through a standardized protocol by a trained technician and data were sent directly through a secured interface to the database, avoiding any data entry mistakes. Finally, statistical analysis was not limited to correlation coefficients calculation, but acknowledged statistical tools for validation and concordance analysis were used [34,36,37,50,51].

In conclusion, this study indicates that Web-based weight and height data from the NutriNet-Santé study can be considered as valid enough to be used when studying associations of nutritional factors with anthropometric and health outcomes. However, underreporting of weight and BMI and overreporting of height was stronger among overweight and obese and we showed misclassification of overweight (sensitivity 87.8%) and obesity (sensitivity 83.3%) which leads us to advise caution when overweight and obesity are the main outcomes. Although it is subject to biases inherent to self-reported anthropometric measurements, the magnitude of such biases can be considered comparable to face-to-face interviews. Therefore, Web-based self-reported data appear to be an accurate and useful tool to assess anthropometric data.

## Multimedia Appendix 1

Statistical analyses for validity and concordance of anthropometrics, NutriNet Santé Study, France, 2012.

## Multimedia Appendix 2

Difference between Web-based self-report and measured anthropometrics according to BMI classification and age class, by sex, NutriNet-Santé study, France 2012.

## Multimedia Appendix 3

Sensitivity analyses among subsample with a time lag between Web-based self-report and measurement < 2 months, NutriNet-Santé study, France, 2012.

## Abbreviations

BMI: body mass index
ICC: intraclass correlation coefficient
LOA: limits of agreement
LTPA: leisure time physical activity
