# Supplementary material for: Validity of Web-Based Self-Reported Weight and Height: Results of the Nutrinet-Santé Study
Source: J Med Internet Res. 2013 Aug 8;15(8):e152. doi: 10.2196/jmir.2575 (PMC3742400; doi:10.2196/jmir.2575)
Supplement: Supplementary file 1 [file jmir_v15i8e152_app1.pdf]

Supplementary table. Statistical analyses for validity and concordance of anthropometrics, NutriNet Santé Study, France, 2012.

VALIDITY (one of the measures is a gold standard)<br>Gold-standard=measured weight/height	CONCORDANCE (between two self-reports)<br>Agreement between web-based and face-to-face self-report
CONTINUOUS VARIABLES: weight, height, BMI	
Difference reported R – measured M:<br>- Paired t-test (on log transformed variables)<br>- Wilcoxon signed rank test of difference	Difference web W - face-to-face F:<br>- Paired t-test (on log transformed variables)<br>- Wilcoxon signed rank test of difference
IntraClass Correlation Coefficient (ICC): measure of agreement between two methods for the same subject. Random effect ICC(2,1) as named by Shrout and Fleiss.<br>Does not take into account the fact that one of the data is the gold standard.	IntraClass Correlation Coefficient (ICC): measure of agreement between W and F for the same subject. Random effect ICC.
Method of Bland and Altman: Plot of the difference R-M on the average (R + M)/2.<br>- Mean difference (requires the normality of differences): if not equal to 0 (or 100% if log transformation), reveals presence of systematic bias :<br>- >0 (or >100%): overreporting, i.e. R>M<br>- <0 (or <100%): underreporting, i.e. R<M<br>- Limits of agreement (LOA) = mean difference ± 2 SD difference. Represents variation interval of the differences for clinical interpretation.<br>- Regression line: Assessment of proportional bias if beta different from zero (CI does not include zero).	Method of Bland and Altman: Plot of the difference W-F on the average (W + F)/2.<br>- Mean difference (requires the normality of differences): if not equal to 0 (or 100% if log transformation), reveals presence of systematic bias :<br>- >0 (or >100%): overreporting W>F<br>- <0 (or <100%): underreporting W<F<br>- Limits of agreement (LOA) = mean difference ± 2 SD difference. Represents variation interval of the differences for clinical interpretation.<br>- Regression line: Assessment of proportional bias if beta different from zero (CI does not include zero).
Multivariate linear regression analyses differences [R-M] as the dependent variable and potential confounders as independent variables (age, sex, smoking status, corpulence, physical activity, education, income, occupation).	
CATEGORICAL VARIABLES: BMI classification (normal weight, overweight	

excluding obesity, obese)	
Percentage of agreement between R and M BMI classification.	Percentage of agreement between W and F reported BMI classification.
Weighted kappa	Weighted kappa
BINARY VARIABLES : overweight including obesity (yes/no), obese (yes/no)	
Sensitivity and Specificity:<br>Truth (gold standard) = measured anthropometric.<br>Sensitivity= TP/(TP+FN)<br>Specificity= TN/(TN+FP)	
Mc Nemar Chi square test : tests if the difference in classification is significant	McNemar Chi square test : tests if the difference in classification is significant

Abbreviations: CI, Confidence Interval; F, face-to-face; FN, False Negatives; FP, False Positives; LOA, Limits of Agreement; M, measured; R, reported; TP, True Positives; TN, True Negatives; W, web-based;
