# Supplementary material for: Validity of Web-Based Self-Reported Weight and Height: Results of the Nutrinet-Santé Study
Source: J Med Internet Res. 2013 Aug 8;15(8):e152. doi: 10.2196/jmir.2575 (PMC3742400; doi:10.2196/jmir.2575)
Supplement: Supplementary file 2 [file jmir_v15i8e152_app2.pdf]

Supplemental Table 2. Difference between web-based self-report and measured anthropometrics according to BMI classification and age class, by sex, NutriNet-Santé study, France 2012.

	Men n=678			Women n=1835		
	Difference web-based - measured			Difference web-based - measured		
	Weight (kg) [a]	Height (cm) [a]	BMI (kg/m²) [a]	Weight (kg) [a]	Height (cm) [a]	BMI (kg/m²) [a]
**Total**	-0.35 ± 1.43	0.61 ± 1.40	-0.31 ± 0.66	-0.47 ± 1.38	0.55 ± 2.66	-0.33 ± 1.66
**BMI classification**						
Normal	-0.11 ± 1.30	0.31 ± 1.29	-0.12 ± 0.50	-0.39 ± 1.30	0.43 ± 1.41	-0.26 ± 0.61
Overweight	-0.68 ± 1.37	0.85 ± 1.32	-0.49 ± 0.62	-0.75 ± 1.48	0.67 ± 4.04	-0.41 ± 3.00
Obese	-0.75 ± 2.05	1.17 ± 1.87	-0.69 ± 1.09	-0.93 ± 1.79	1.10 ± 4.67	-0.74 ± 2.45
p-value [b]	<.0001	<.0001	<.0001	.004	.03	.0006
**Age**						
18-25y	-0.60 ± 2.07	0.00 ± 2.35	-0.28 ± 0.70	-0.42 ± 1.38	0.27 ± 0.98	-0.23 ± 0.59
25-35y	0.02 ± 1.65	0.25 ± 1.43	-0.07 ± 0.69	-0.36 ± 1.57	0.22 ± 1.25	-0.19 ± 0.68
35-45y	-0.20 ± 1.25	0.31 ± 1.25	-0.15 ± 0.52	-0.55 ± 1.52	0.27 ± 1.186	-0.29 ± 0.75
45-55y	-0.29 ± 1.53	0.29 ± 1.31	-0.19 ± 0.61	-0.54 ± 1.34	0.37 ± 3.20	-0.28 ± 1.65
55-65y	-0.44 ± 1.41	0.63 ± 1.30	-0.35 ± 0.66	-0.54 ± 1.43	0.76 ± 1.58	-0.45 ± 0.76
>=65y	-0.54 ± 1.44	0.88 ± 1.50	-0.45 ± 0.69	-0.58 ± 1.29	0.83 ± 4.52	-0.38 ± 3.37
p-value [c]	.15	.0006	.0002	.80	.04	.05

[a] Values are mean ± SD
[b] p-value of the effect of BMI category on the log-transformed difference
[c] p-value of the effect of age category on the log-transformed difference
