# Supplementary material for: Validity of Web-Based Self-Reported Weight and Height: Results of the Nutrinet-Santé Study
Source: J Med Internet Res. 2013 Aug 8;15(8):e152. doi: 10.2196/jmir.2575 (PMC3742400; doi:10.2196/jmir.2575)
Supplement: Supplementary file 3 [file jmir_v15i8e152_app3.pdf]

Supplemental Table 3. Sensitivity analyses among subsample with a time lag between web-based self-report and measurement < 2 months, NutriNet-Santé study, France, 2012

**Validity n=2 079**													
	Web-based		Measured		Agreement (%)		Weighted Kappa[a]			Sensitivity [c,d]		Specificity [c,e]	
	n	%	n	%	%	95% CI	κ	95% CI	*P* [b]	Se	95% CI	Spe	95% CI
**BMI classification**					95.0	94.1-95.9	0.92	0.91 - 0.94					
Normal (BMI<25)	1387	66.71	1377	66.23									
Overweight (BMI 25-29.9)	478	22.99	484	23.28					.30	0.91	0.89 - 0.93	0.99	0.98 - 1.0
Obese (BMI≥30)	214	10.29	218	10.49					.57	0.91	0.87 - 0.95	1.00	0.99 - 1.0
	Web-based		Measured		Difference						ICC [g]		
	Mean	SD	Mean	SD	Mean	SD	*P* [f]			ICC	95% CI		
Weight (kg)	67.13	14.17	67.76	14.38	-0.62	-0.72	<.0001			0.988	0.987-0.989		
Height (cm)	166.81	8.46	166.26	8.45	0.55	0.49	<.0001			0.984	0.983-0.986		
BMI (kg/m^2)	24.19	4.56	24.22	4.52	-0.03	-0.06	.01			0.986	0.985-0.987		

**Concordance n=233**											
	Web-based		Face to face		Agreement (%)		Weighted Kappa[a]				
	n	%	n	%	%	95% CI	κ	95% CI	*P* [b]		
**BMI classification**					94.0	90.9-97.0	0.87	0.82 - 0.92			
Normal (BMI<25)	141	60.52	149	63.95							
Overweight (BMI 25-29.9)	69	29.61	64	27.47					.18		
Obese (BMI≥30)	23	9.87	20	8.58					.38		
	Web-based		Face to face		Difference						ICC [g]
	Mean	SD	Mean	SD	Mean	SD	*P* [f]			ICC	95% CI
Weight (kg)	66.26	12.56	65.94	12.50	0.33	1.71	.01			0.990	0.986-0.993
Height (cm)	165.91	7.74	165.87	7.87	0.03	0.87	.54			0.993	0.991-0.995
BMI (kg/m^2)	24.19	4.19	23.92	4.05	0.27	0.75	<.0001			0.981	0.975-0.985

[a] Cicchetti-Allison weight. For a given cell in row i, colon j, Wij=1-|i-j|/2

[b] *P*-value of McNemar χ^2 test for binary variables: overweight including obesity (BMI>=25) yes/no and obese (BMI>=30) yes/no. A p-value lower than 0.05 indicates significant difference between web-based self-reporting and measurement.

[c] Sensitivity and Specificity for binary variables: overweight including obesity (BMI>=25) and obese (BMI>=30).

[d] Sensitivity=True Positives/(True positives + False negatives).

[e] Specificity=True Negatives / (True Negatives + False positives). Truth = clinical data. SD=$[p(1-p)/n]^{1/2}$ Confidence interval is calculated as sensitivity±1.96*SD

[f] P-value of the paired t-test of difference of log-transformed variable ("Web minus face-to-face")

[g] ICC(2,1) calculated on log transformed variables
